# A retrospective analysis of maternal and neonatal mortality at a teaching and referral hospital in Kenya

**DOI:** 10.1186/1742-4755-10-13

**Published:** 2013-02-19

**Authors:** Faith Yego, Jennifer Stewart Williams, Julie Byles, Paul Nyongesa, Wilson Aruasa, Catherine D'Este

**Affiliations:** 1 Department of Health Policy and Management, Moi University, Nandi Road, Eldoret 30100, Kenya; 2 Research Centre for Gender, Health and Ageing, HMRI Building, University of Newcastle, University Drive, 2308, Callaghan, NSW, Australia; 3 Reproductive Health Department, Moi University, Nandi Road, 30100, Eldoret, Kenya, Australia; 4 Clinical Services, Moi Teaching and Referral Hospital, Nandi Road, 30100, Eldoret, Kenya; 5 Centre for Clinical Epidemiology and Biostatistics, HMRI Building, University of Newcastle, University Drive, 2308, Callaghan, NSW, Australia

**Keywords:** Maternal mortality, Early neonatal mortality, Determinants, Referral hospital, Kenya, Maternal mortality ratio, Early neonatal mortality rate

## Abstract

**Objective:**

To measure the incidence of maternal and early neonatal mortality in women who gave birth at Moi Teaching and Referral Hospital (MTRH) in Kenya and describe clinical and other characteristics and circumstances associated with maternal and neonatal deaths following deliveries at MTRH.

**Methods:**

A retrospective audit of maternal and neonatal records was conducted with detailed analysis of the most recent 150 maternal deaths and 200 neonatal deaths. Maternal mortality ratios and early neonatal mortality rates were calculated for each year from January 2004 to December 2011.

**Results:**

Between 2004 and 2011, the overall maternal mortality ratio was 426 per 100,000 live births and the early neonatal mortality rate (<7 days) was 68 per 1000 live births. The Hospital record audit showed that half (51%) of the neonatal mortalities were for young mothers (15–24 years) and 64% of maternal deaths were in women between 25 and 45 years. Most maternal and early neonatal deaths occurred in multiparous women, in referred admissions, when the gestational age was under 37 weeks and in latent stage of labour. Indirect complications accounted for the majority of deaths. Where there were direct obstetric complications associated with the delivery, the leading cause of maternal death was eclampsia and the leading cause of early neonatal death was pre-mature rupture of membranes. Pre-term birth and asphyxia were leading causes of early neonatal deaths. In both sets of records the majority of deliveries were vaginal and performed by midwives.

**Conclusion:**

This study provides important information about maternal and early neonatal mortality in Kenya’s second largest tertiary hospital. A range of socio demographic, clinical and health system factors are identified as possible contributors to Kenya’s poor progress towards reducing maternal and early neonatal mortality.

## Background

In developing countries, more than nine million infants die every year before birth and in the first week of life as a result of complications occurring during pregnancy. Many of these deaths are preventable [1]. In 2000 the United Nations (UN) made a declaration to include maternal and child mortality reduction as a target in its Millennium Development Goals (MDGs) [2]. Maternal mortality is high throughout Africa, yet the ratios are particularly high in Kenya, where a woman’s lifetime risk of dying is one in 38 compared to one in 2000 in the developed world [1]. The World Health Organization (WHO) reported that Kenya’s progress towards improving maternal and neonatal health is presently “insufficient” with little or no progress having been made over the past decade [3].

Of the more than 500,000 women who die each year as a result of complications arising during pregnancy, half live in Sub-Saharan Africa [4]. Yet death is not the only outcome resulting from pregnancy complications. For every woman who dies, at least 30 others are injured and disabled. Globally seven million women are affected by health problems related to childbearing [5]. Despite the inauguration of the Safe Motherhood Initiative (SMI) in Nairobi in 1987, Kenya has made limited progress towards improving maternal mortality.

Between 1980 and 2010, the national maternal mortality ratio (MMR) was 400–560 per 100,000 live births [1,6,7]. The ratios are higher for the major teaching and referral hospitals where obstetrics complications are concentrated. For example, the MMR in Kenya’s largest referral hospital, Kenyatta National Hospital (KNH), was 922 per 100,000 live births in 2004 [8]. In Kilifi District Hospital in Kenya, the MMR was 250 per 100,000 live births between 2008 and 2010 [9]. In nearby Sub-Saharan African countries, MMRs in teaching hospitals are also high. For instance, in Adeoyo Hospital in Nigeria, the MMR was 963 per 100,000 live births between January 2003 and December 2004 [10]. The Neonatal Mortality Rate (NMR) in KNH from January to December of 2000 was 215 per 1000 live births [11]. The NMRs are high in other African countries such as Nigeria, (53 per 1000 live births) and Ethiopia (51 per 1000 live births) [12].

Newborn deaths represent 38% of all deaths among children under five years of age [13]. One in five women in Africa risks losing a newborn baby during her lifetime [14]. Pre-term birth accounts for 29% of neonatal deaths globally and approximately 14% of babies are born with low birth weight [13]. Early neonatal outcomes can be affected by nutrition, lifestyle and socio-economic status of mothers. “The best care in the world cannot save a woman’s life if she cannot reach it, cannot afford it, does not know it is there when to seek it, or is not permitted to use it” [15].

The Delay Model by Thaddeus and Maine [16,17] provides a suitable conceptual framework for understanding risk factors associated with maternal mortality at a tertiary referral hospital. The Model identifies three types of delays. They are: delay in the decision to seek care, delay in arrival at a health facility and delay in the provision of adequate care [16]. Some risk factors that have been linked to the delay model are: lack of funding, inaccessibility, poor infrastructure, inadequate staffing, inadequate equipment and supplies, lack of information, cultural issues, social vulnerability, and low socio-economic status [17].

Ensuring the continuum of care throughout pregnancy is an important requirement for the reduction of maternal and early neonatal deaths. There is evidence that a significant number of stillbirths and neonatal deaths could be prevented if all women were adequately nourished and received good quality care during pregnancy, delivery, and the postpartum period [14,18]. The antenatal period helps the health care provider to assess risks and treat conditions that could affect both the mother and baby [19]. It is essential that during delivery, obstetric emergencies are effectively managed to prevent complications which account for up to 58% of stillbirths and early neonatal deaths [20]. Countries such as Thailand, Sierra Leonne, Libera, Pakistan, Sudan, Bosnia, Uganda, Tanzania, and Northern Kenya, have established intervention projects to improve the availability of emergency obstetric care (EmOC) [21]. These projects include the use of signal funtions to assess whether their health facilties adhere to international standard operating procedures for the management of emergencies during pregnancy.

In the post-partum period, the provision of family planning advice after delivery is of vital importance, especially in settings where the birth rate is high and multiparous women are at repeated risk of pregnancy complications and adverse birth outcomes [19].

Health care infrastructure in Kenya includes two national tertiary teaching and referral hospitals as well as provincial hospitals, district and sub-district hospitals, health centres and dispensaries, or chemists. The private sector provides about one third of outpatient care and 14% of inpatient care. High-risk patients are managed in the tertiary hospitals where clinical resources are more specialized. A number of measures have been introduced to help meet the MDGs in Kenya. For example, Kenya's second largest referral hospital, the Moi Teaching and Referral Hospital (MTRH) has initiated 24 hour maternal and perinatal death reviews and monthly maternal mortality reviews for all maternal deaths. Over the past two years the MTRH has also established standard operating procedures for managing both direct and indirect maternal complications in pregnancy. However more work is needed to achieve progress in this area.

While there have been many maternal health studies in Kenya, little has been published specifically on maternal and early neonatal mortality [8,11,22-26]. The aim of this study is to measure the incidence of maternal and neonatal mortality in women who gave birth at MTRH and describe clinical characteristics and circumstances associated with maternal and early neonatal deaths following deliveries at MTRH. As one of only two teaching and referral hospitals in Kenya, the MTRH serves an important role in the country’s health system. MTRH is also the largest hospital in the rural western region of Kenya. High maternal and early neonatal mortality at MTRH has local and national implications and therefore requires investigation.

## Methods

A retrospective audit of maternal and neonatal records at MTRH between January 2004 and March 2011 was conducted. Detailed information was independently extracted by trained abstractors. The sample included the most recently hospitalized 150 women, aged 15–49 years, who were classified as maternal deaths, and the most recently hospitalized 200 neonates, who died after delivery or within seven days of delivery. Record numbers were based on the sample size needed for a subsequent case control study of maternal and early neonatal deaths at MTRH. This was obtained by assuming the probability of exposure was 40% and the ratio of deaths to survivors was 1:2. A sample of approximately 450 women (150 cases and 300 controls) was sufficient to detect an absolute difference in risk factor prevalence of at least 15% (80% power, 95% significance). These calculations were made using PS power software. Standard definitions of maternal mortality, early neonatal mortality, neonatal mortality, and direct causes of death were used [27,28]. Non-pregnancy related deaths were not included.

### Data source and setting

The MTRH is located in Kenya’s Rift Valley province [29] providing a range of curative, preventive and rehabilitative services. The catchment covers a population of over seven million inhabitants [6] and the MTRH also accepts referrals from Kenya’s 13 million indigent population in the north and west [29]. The reproductive health department at MTRH has 17 obstetrician-gynaecologists, five medical officers, two clinical officers, 100 nurses who are either trained midwives or have basic training in midwifery [29]. The reproductive department at MTRH has four medical wards, and an obstetric mother/baby unit with a capacity of 150 beds [29].

Patients at MTRH are referred either from other hospitals or the community, usually following evidence of complications [29]. Deliveries at MTRH occur in the labour wards where women are delivered in maternity couches and mostly attended by midwives, but in cases of complications, attended by doctors [29]. The Hospital provides gloves and linen necessary for deliveries [29].

### Descriptive variables

Information extracted from mothers’ hospital records included: age, parity, gestational age, maternal complication on admission, pregnancy stage, stage of labour, birth attendant at delivery, and booking status. Patients who were referred i.e. from lower level clinical facilities or self referred from home or by a traditional birth attendant, were classified here as “unbooked”. All other patients who attended antenatal clinics at MTRH and had been scheduled to deliver at MTRH were classified as “booked”.

Information extracted from neonates’ hospital records included: outcome at birth, apgar score, birth weight, gender, complication at birth, and mothers’ and neonates’ condition at discharge. All definitions of causes of death were based on the WHO International Classification of Diseases Version 10 (ICD-10). Where multiple causes of death were recorded, the primary cause was identified using available documentation and post mortem reports.

### Statistical analyses

The retrospective audit at MTRH, which covered the period January 2004 to March 2011, provided descriptive information on mothers and babies. This was determined by data available at the time the study was undertaken. The annual incidence estimates were augmented by data provided by the records department at the MTRH, giving total numbers of live births and maternal and early neonatal deaths per year between 1^st^ January 2004 and 31^st^ December 2011. These data were used to calculate the annual maternal mortality ratios and early neonatal mortality rates. STATA version 11 was used for all statistical analyses. The study was approved by the Human Resource and Ethics Committee at University of Newcastle, Australia, and the Institute of Research and Ethics Committee at Moi University and MTRH.

## Results

**Figure 1:**
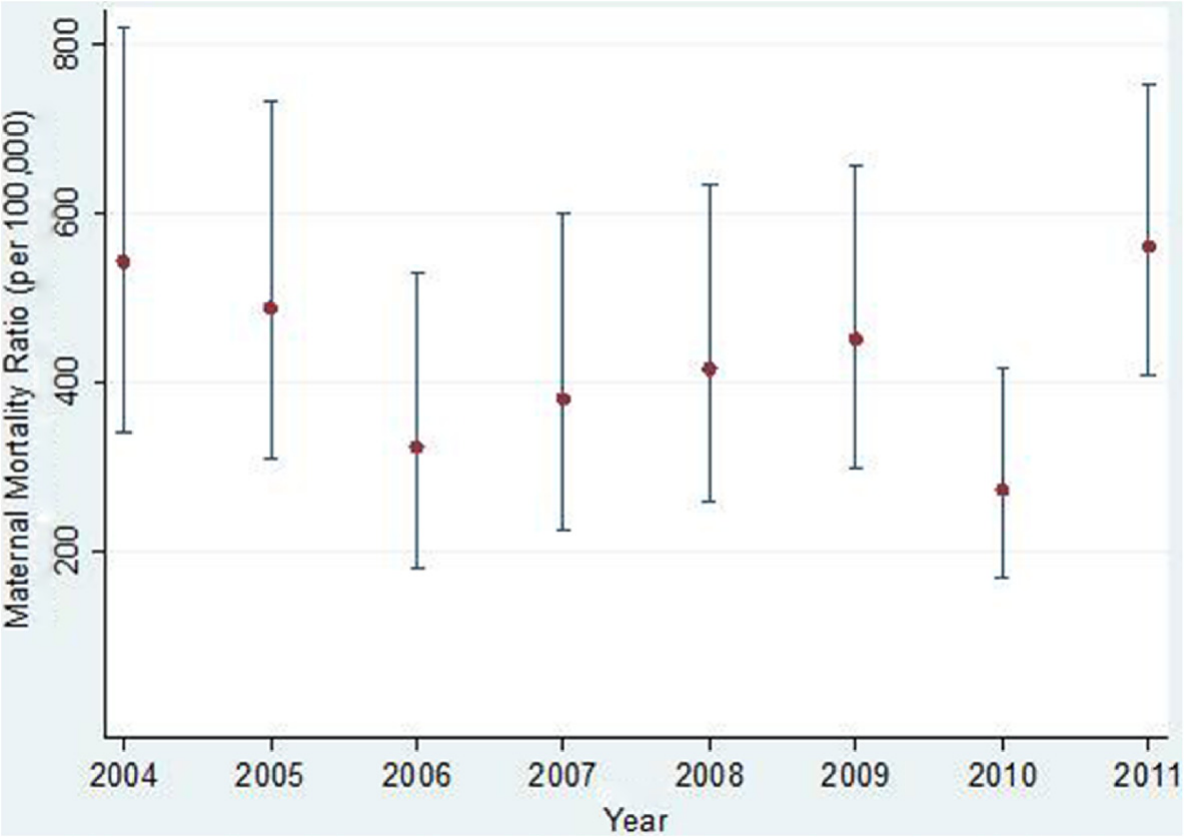
**Annual maternal mortality ratios with 95% confidence intervals at MTRH from January 2004 to December 2011. **Source: MTRH records department.

**Figure 2:**
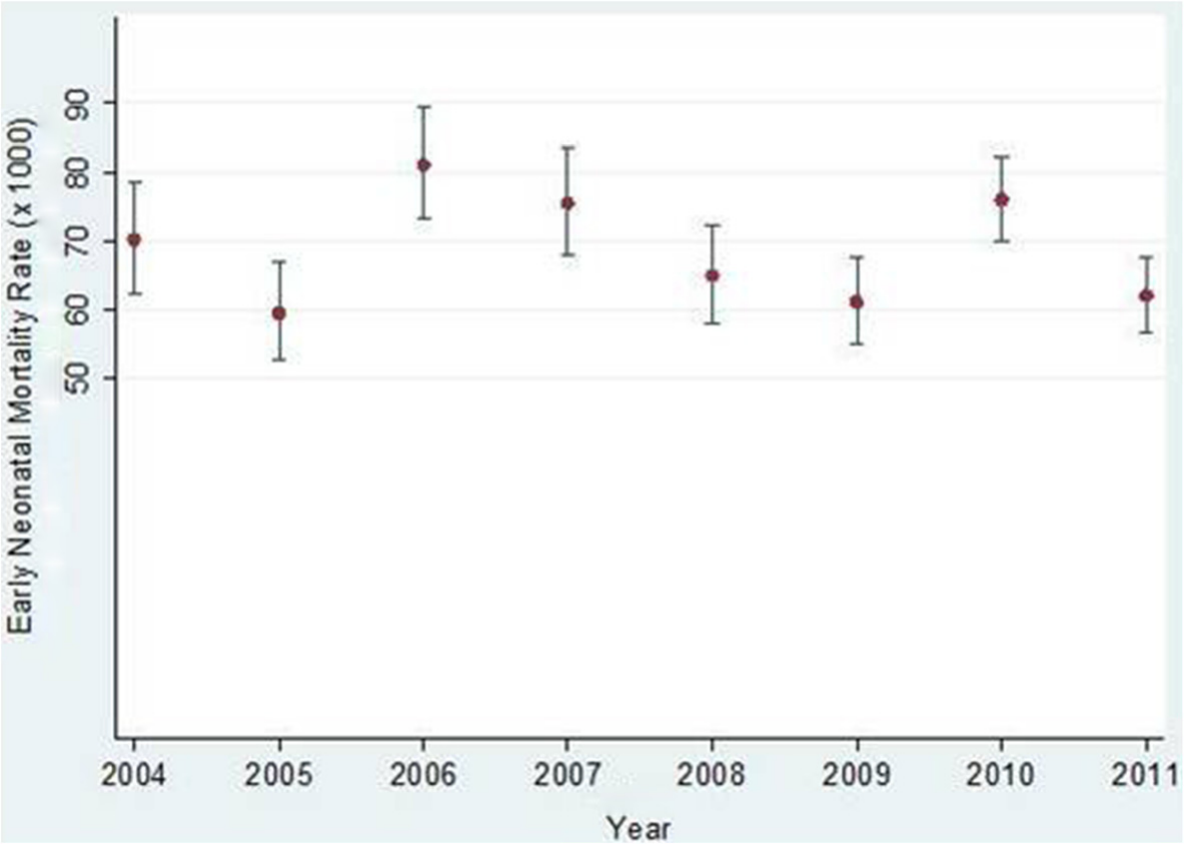
Annual neonatal mortality rates with 95% confidence intervals at MTRH from January 2004 - to December 2011.

Figures 1 and 2 show annual changes in the MMRs and NMRs with 95% confidence intervals. The overall MMR was 426 per 100,000 live births and the overall NMR was 68 per 1,000 live births. There were wide variations between 2004 and 2011. For example in 2010, the MMR was the lowest and the NMR was second highest for the period. Despite different point estimates, year-to-year differences in maternal mortality were not statistically significant as seen by the overlapping 95% confidence intervals. However neonatal mortality was significantly different between 2005 and 2006, 2009 and 2010, and 2010 and 2011.

Table 1 shows the maternal and obstetric characteristics for the 150 maternal deaths and the 200 early neonatal deaths. Half (51%) of the early neonatal mortalities were for young mothers (15–24 years), and 64% of maternal deaths occurred in women aged between 25 and 45 years. For both the maternal and early neonatal deaths, high proportions (49% and 54% respectively) of mothers were multigravid. The majority of birth attendants were midwives for both maternal and early neonatal deaths (53% and 71% respectively). The majority of mothers with early neonatal deaths (56%) were admitted at the intrapartum stage of pregnancy (which is the period from start of labour to delivery). A high proportion of mothers who died (42%) were also admitted at the intrapartum stage. The labour stage at admission was mostly active (87%) for the neonatal deaths. The labour stage for the maternal deaths was mostly latent (42%). This is the early or slow phase of labour. The gestational age was commonly less than 36 weeks. A high proportion of these deliveries were vaginal (43% for the maternal deaths and 73% for the neonatal deaths). Most mothers (58%) were not pre-booked at the MTRH but referred from home and other facilities, and most neonates (79%) were referrals.

**Table 1:** Maternal and obstetric characteristics of maternal and early neonatal deaths at MTRH from January 2004 to March 2011

**Characteristic**	**Maternal deaths n = 150**		**Neonatal deaths n = 200**	
	**n**	**%**	**n**	**%**
**Age (years)**				
15-24	54	36	97	51
25-34	66	44	68	35
35-45	30	20	27	14
**Gravida**				
Primigravida (1)	34	23	58	29
Multigravida (2–4)	73	49	108	54
Grandmultigravida (5–7)	32	21	26	13
Grandgrandmultigravida (>8)	11	7	8	4
**Birth attendant at delivery**				
Doctor	61	47	50	26
Midwife	70	53	136	71
**Pregnancy stage on admission**				
Antepartum	53	37	73	37
Intrapartum	61	42	110	56
Puerperium	30	21	14	7
**Labour stage on admission**				
Latent	42	42	16	9
Active	37	37	156	87
Second stage	20	20	7	4
**Gestational age**				
<36 weeks	78	59	116	58
37-41 weeks	37	28	39	19
<42 weeks	3	2	5	2
Post partum	14	11	40	20
**Mode of delivery**				
Vaginal	64	43	146	73
Operation	51	34	45	22
Assisted delivery	27	18	9	5
Other	8	5	0	0
**Booking status**				
Booked	63	42	38	21
Unbooked (referral)	87	58	139	79

Denominators vary for each item depending on missing data.

The highest number of deliveries by a single mother at the MTRH was 13.

Pregnancy complications associated with maternal and early neonatal deaths at MTRH are shown in Table 2. Eclampsia (22%) was the leading direct complication for maternal death, followed by dystocia (14%), and hemorrhage (13%). For neonatal deaths the leading maternal complication was premature rupture of the membrane (PROM) (26%) followed by dystocia (22%). Table 3 shows that the leading neonatal complications among the maternal and early neonatal deaths were asphyxia (17% of maternal deaths and 25% of early neonatal deaths) and pre-term birth (13% of maternal deaths and 38% of early neonatal deaths).

**Table 2:** Pregnancy complications for maternal and early neonatal deaths at MTRH from January 2004 to March 2011

**Pregnancy complication**	**Maternal deaths n = 150**		**Neonatal deaths n = 200**	
	**n**	**%**	**n**	**%**
Eclampsia	33	22	13	7
Dystocia	21	14	44	22
Hemorrhage	20	13	17	8
Sepsis	10	7	0	0
Post abortal	10	7	5	3
Premature rupture of membrane (PROM)	2	1	52	26
Post datism	1	1	3	1
Other (indirect)†	53	35	66	33

Denominators vary for each item depending on missing data.

†Indirect causes include: HIV, malaria and cardiovascular diseases.

**Table 3:** Neonatal complications for maternal and early neonatal deaths at MTRH from January 2004 to March 2011

**Neonatal complication**	**Maternal deaths n = 150**		**Neonatal deaths n = 200**	
	**n**	**%**	**n**	**%**
None	84	56	0	0
Pre-term birth	16	13	75	38
Asphyxia	20	17	51	25
Sepsis	1	1	29	14
Congenital malformation	2	2	21	11
Other	12	10	24	10

Denominators vary for each item depending on missing data.

Neonatal outcomes for both maternal and early neonatal deaths at MTRH are given in Table 4. Among the neonatal deaths, majority of the neonates (86%) were alive at birth. Among the maternal deaths, 45% of the neonates were alive at birth but only 25% were discharged alive. There was a high proportion of missing information for the neonates born to mothers who died (e.g. weight 55%, gender 38%, and apgar score 39%). Amongst neonates, the apgar score had the highest proportion of missing information (21%).

**Table 4:** Early neonatal and maternal outcomes at MTRH from January 2004 to March 2011

**Outcome**	**Maternal deaths n = 150**		**Neonatal deaths n = 200**	
	**n**	**%**	**n**	**%**
**Baby’s outcome at birth**				
Alive	66	45	172	86
Stillbirth	61	41	28	14
Early neonatal death	20	14	0	0
Missing	4	2	0	0
**Baby’s weight at birth**				
500-2499gms	31	20	133	67
2500-4499gms	36	24	49	25
Missing	82	55	18	9
**Apgar score at 5 mins**				
0-6	61	41	78	39
7-10	31	20	79	40
Missing	58	39	43	21
**Baby’s gender at birth**				
Male	41	27	105	52
Female	52	35	85	43
Missing	57	38	10	5
**Baby’s condition on discharge**				
Alive	38	25	0	0
Neonatal death	96	64	200	100
Missing	15	10	0	0
**Mothers condition on discharge**				
Alive	0	0	186	98
Death	150	100	4	2
Missing	0	0	0	0

Denominators vary for each item depending on missing data.

## Discussion

This study provides important information about maternal and early neonatal mortality in Kenya’s second largest tertiary hospital. The MTRH draws referrals from a large catchment area and a high proportion of admissions are for women with obstetric complications. By conducting a secondary analysis of records in this large tertiary referral Hospital with high-risk obstetrics admissions, we were able to measure annual ratios for maternal mortality and early neonatal mortality rates in women who gave birth at MTRH, and describe clinical and other characteristics and circumstances surrounding maternal and early neonatal deaths following deliveries at MTRH. It is intended that this information will be used to change policies and practices that will lead to improvements in maternal and early neonatal mortality.

The findings are in agreement with other studies in developing countries in which, like Kenya, progress in reducing maternal and early neonatal mortality has been slow [30,31]. The MMR and NMR are best estimates based on available data from a major referral hospital. The peaks in maternal and early neonatal mortality at MTRH in 2011 and 2010 may be explained in part by industrial strikes at the Hospital during this time. The strikes reduced staffing levels placing pressure on Hospital resources at a time when birth numbers were fairly high. Birth rates have increased in Kenya over the past decade leading to a tripling in the population [6]. These trends may also have contributed to the higher mortality ratios seen here.

The study found that half (51%) of the early neonatal mortalities were for younger mothers (15–24 years) and 64% of maternal deaths were in women aged between 25 and 45 years. Evidence from other studies in developing countries also shows that high proportions of early neonatal deaths occur among teenage mothers and that maternal deaths occur among women who are multigravid [32-35]. In this study most maternal and early neonatal deaths occurred for multiparous women and in unbooked women whose gestational age was under 37 weeks. Mortality occurred among women who were admitted in the latent stage of labour (42%). This could be due to, for example, delayed labour ward admission, and lack of strict criteria for admission into labour wards [36].

Indirect obstetric complications accounted for about one third of the maternal and early neonatal deaths, with direct complications accounting for two thirds, possibly reflecting poor diagnosis and treatment of diseases that developed during pregnancy. However, other studies have shown that direct pregnancy complications contribute to a higher proportion of maternal deaths than indirect complications [3,37,38]. In this study the majority of maternal and early neonatal deaths were among women whose babies were at lower gestational age. Lower gestational age increases risk of death [33,34] and other studies have reported similar findings. Babies born under 37 weeks gestation are at higher risk of pre-mature birth and hence adverse birth outcomes [3,12,32].

Compared with assisted or caesarean delivery, the majority of the maternal and early neonatal deaths followed vaginal deliveries. Studies show that maternal and early neonatal deaths are associated with the mode of delivery and also medical practices. For example, some doctors may be unwilling to intervene aggressively on behalf of the fetus [33]. Access to skilled birth attendants (including doctors and midwives) is essential for the prevention of maternal and early neonatal deaths and this is still an issue in Sub-Saharan Africa. In Kenya, the majority of deliveries are managed by traditional birth attendants in the communities. Many such attendants lack appropriate skills which can contribute to maternal and early neonatal morbidities and mortalities.

Of the maternal and early neonatal deaths at MTRH, more than half were referred admissions. Studies in Africa have shown that of the women who are referred to hospitals for deliveries, many have severe or life threatening complications [10]. There is evidence that newborn deaths are higher in cases where best practice for newborn care is limited [12,35].

While there are a number of areas that could be followed up, some key points are noted here. There is a need to improve hospital referral policies, and also review clinical guidelines and management protocols for at-risk mothers. There is also need for attendance at antenatal clinics in order to screen for underlying illnesses and ensure proper management of complications that can occur in pregnancy.

This work has strengths and limitations. This is the first study of its kind to be conducted at a major tertiary teaching and referral hospital in Kenya. The MTRH allowed access to individual patient records. This provided a means of describing the characteristics and circumstances surrounding maternal and early neonatal deaths associated with deliveries at MTRH. Importantly this study provides a platform for identifying a range of issues that can be addressed in future efforts to reduce maternal and early neonatal deaths in other similar hospitals. The fact that detailed hospital level data were analysed also makes it possible to suggest changes in hospital policies, practices and procedures that may ultimately reduce maternal and early neonatal mortality. Although undertaken from a hospital perspective, the work contributes more generally to understanding some of the reasons for Kenya’s lack of progress towards achieving MDGs 4 and 5 by 2015.

A possible limitation is that the work is not generalizable at a national level. The data comprise only hospital births. Both nationally and in the MTRH catchment, approximately 40-43% of births are in hospitals. The proportion is similar in the MTRH catchment area [6]. A second limitation was the difficulty in estimating the MMR and NMR due to small numbers in the denominators, as evident from the overlapping confidence intervals. A further limitation is that a high proportion of the medical records collected for the study were incomplete or had missing data. It was impossible to say how much data were missing, and it is not known to what extent the missing data may have biased results. The huge proportion of missing data in neonatal variables highlights the need to link maternal and neonatal records so that information can be easily retrieved for both mothers and their babies, especially when there are adverse outcomes.

## Conclusion

Maternal and early neonatal mortality remains high in Kenya despite the efforts to achieve MDGs four and five. Using data collected in a large tertiary referral hospital, this descriptive study identified a range of socio demographic, clinical and health system factors as possible contributors to Kenya’s poor progress towards reducing maternal and early neonatal mortality. Further research is needed in order to understand other possible contributors, such as those found in the community, and factors associated with quality of care.

## Consent

Written and informed consent was obtained for publication of this report and any accompanying images.

## Abbreviations

MTRH: Moi Teaching and Referral Hospital; UN: United Nations; WHO: World Health Organization; MDGs: Millennium Development Goals; SMI: Safe Motherhood Inititative; ICD: International Classification of Diseases; NMR: Neonatal Mortality Rate; MMR: Maternal Mortality Ratio; PROM: Premature rupture of membranes; EmOC: Emergency Obstetric Care

## Competing interests

The authors declare that they have no competing interests.

## Authors’ contributions

FY participated in all stages of the study including design, implementation, data collection, analysis and writing. JSW contributed input to the study design, analysis and interpretation, and assisted in drafting and editing the manuscript. JB contributed to the study design and provided intellectual input at all stages of the research. PN assisted with the design of the study, data collection, interpretation and manuscript preparation. WA provided input regarding study design and data interpretation. CD advised on all statistical issues and also provided intellectual input at all stages of the study. All authors read and approved the final manuscript.
